# The coexistence of stunting and overweight or obesity in Ethiopian children: prevalence, trends and associated factors

**DOI:** 10.1186/s12887-023-04037-7

**Published:** 2023-05-05

**Authors:** Biniyam Sahiledengle, Lillian Mwanri, Abera Kumie, Girma Beressa, Daniel Atlaw, Yohannes Tekalegn, Demisu Zenbaba, Fikreab Desta, Chala Kene, Kenbon Seyoum, Degefa Gomora, Demelash Woldeyohannes, Kingsley Emwinyore Agho

**Affiliations:** 1 Department of Public Health, Madda Walabu University Goba Referral Hospital, Bale-Goba, Ethiopia; 2 Centre for Public Health Research, Equity and Human Flourishing, Torrens University, Adelaide Campus, Adelaide, SA 5000 Australia; 3 School of Public Health, College of Health Science, Addis Ababa University, Addis Ababa, Ethiopia; 4 Department of Human Anatomy, Madda Walabu University Goba Referral Hospital, Bale-Goba, Ethiopia; 5 Department of Midwifery, Madda Walabu University Goba Referral Hospital, Bale-Goba, Ethiopia; 6 Department of Public Health, College of Medicine and Health Science, Wachemo University, Hossana, Ethiopia; 7 School of Health Sciences, Western Sydney University, Locked Bag 1797, Penrith, NSW 2751 Australia

**Keywords:** Double burden, Malnutrition, Concurrent stunting and overweight or obesity, Ethiopia

## Abstract

**Background:**

Double burden of childhood malnutrition is a condition where undernutrition (stunting) along with overweight and obesity coexist within individuals, households, and populations. It reflects a new layer of malnutrition and an understudied phenomenon in many low-income settings. To date, the prevalence and factors that are associated with concurrent stunting and overweight or obesity (overweight/obesity) (CSO) in the same children have not been well researched in Ethiopia. Hence, this study aimed to assess the prevalence, trends, and factors associated with the coexistence of stunting and overweight or obesity among children aged 0–59 months in Ethiopia.

**Methods:**

Pooled data from 2005, 2011 and 2016 Ethiopian Demographic and Health Survey (EDHS) were used. A total of 23,756 (weighted sample) children aged 0–59 months were included in the study. Height-for-age z-scores (HAZ) less than − 2 SD and weight-for-height z-scores (WHZ) above 2 SD were calculated, and children were classified as stunted and overweight/obese, respectively. A child who is simultaneously stunted and overweight/obese was considered as having HAZ below − 2 SD and WHZ above 2 SD computed into a variable named CSO, and reported as a binary outcome (yes or no). Multilevel logistic regression analysis that adjusts for sampling weights and clustering was used to identify factors associated with CSO.

**Results:**

The prevalence of stunting, overweight or obesity, and CSO among under-five children was 43.12% [95% CI: (42.50, 43.75%)], 2.62% [95% CI: (2.42, 2.83%)], and 1.33% [95% CI: (1.18, 1.48%)], respectively. The percentage of CSO children was reported to have declined from 2.36% [95% CI: (1.94–2.85)] in 2005 to 0.87% [95%CI: (0.07–1.07)] in 2011, and the same appeared to have increased slightly to 1.34% [95%CI: (1.13–1.59)] in 2016. Children who were currently breastfeeding [AOR: 1.64, 95%CI: (1.01–2.72)], born to an overweight mother [AOR: 2.65, 95%CI: (1.19–5.88)], and lived in families with 1–4 household members [AOR: 1.52, 95%CI: (1.02–2.26)] were significantly associated with CSO. At the community level the odds of having CSO were higher among children included from EDHS-2005 [AOR: 4.38, 95%CI: (2.42–7.95)].

**Conclusion:**

The study revealed that less than 2% of children had CSO in Ethiopia. CSO was linked to factors at both the individual (i.e. breastfeeding status, maternal overweight, and household size) and community-levels. Overall, the study findings indicated the necessity of focused interventions to simultaneously address double burden of childhood malnutrition in Ethiopia. To further combat the double burden of malnutrition, early identification of at-risk children, including those born to overweight women and children living with multiple household members, is indispensable.

**Supplementary Information:**

The online version contains supplementary material available at 10.1186/s12887-023-04037-7.

## Introduction

Childhood malnutrition remains a serious public health challenge in low-income countries. Malnutrition in children refers to deficiencies or excesses in nutrient intake, imbalance of essential nutrients, or impaired nutrient utilization [1]. Children can also experience two contrasting forms of malnutrition, a condition termed the double burden of malnutrition (DBM), which has become a growing global challenge [2–4]. According to the World Health Organization (WHO) the double burden of malnutrition (DBM) is “characterized by the coexistence of undernutrition (stunting) along with overweight/obesity, and may lead to diet-related non-communicable diseases, within individuals, households, and populations, and across the life course” [2, 5]. For DBM to occur at the household level, at least one member in the household may be undernourished (i.e. stunted, wasted, or underweight) and at least one member is overweight/obese [6]. In contrast, at the individual level, the DBM is expressed when an individual is stunted during early life and may be overweight later in life; or an individual may have a co-existence of micronutrient deficiencies with overweight or obesity at the same time [6]. At the individual level, it has increasingly been observed that children can be overweight and stunted simultaneously (CSO) [7]. Evidence indicates that the best-targeted age to address childhood malnutrition is the first 1000 days of life as this window period is ideal for intervention implementation and tracking for the improvement of child growth and development [8].

Stunting is the most prevalent form of childhood growth failure across all years and countries. In 2019, 155 million children under 5 years of age were stunted [1] and 38.2 million were overweight or obese globally [9]. The concurrence of undernutrition and overweight has been increasing in the poorest low and middle-income countries (LMICs) due to changes that have been termed as the nutrition transition [4], resulting from lifestyle preferences, environmental factors, and cultural determinants [10]. In Africa, the number of overweight children has increased by nearly 24% since 2000 [9]. Conversely, stunting prevalence reduced from 34.5 to 31.1% between 2012 and 2019, but not sufficiently enough to reach the worldwide target [11]. The average prevalence of stunting in sub-Saharan Africa (SSA) was estimated to be 41% [12]. In the Eastern African region where Ethiopia is located, stunting continues to be a rampant public health concern. The region also bears the greatest proportion of under-five stunting and overweight or obese reported be 33.3% [13] and 4.59% [14], respectively.

Studies in different LMICs so far have focused on DBM of various forms of malnutrition in mother–child pairs residing in the same household [15–17]. Some studies have also investigated the co-morbid anemia and stunting among children aged 6–59 [18–21], and others have focused on concurrent stunting and wasting [22–25]. However, few studies have focused on the co-occurrence of stunting and overweight/obesity (CSO) in the same children [7, 26–30]. For instance, Fongar et al. (2019) conducted a study on the coexistence of overweight/obesity and undernutrition in under-fives in western Kenya and reported the prevalence of DBM to be 1.1% [27]. In a Vietnamese study that CSO was reported to be 2.7% in 2013 and 1.4% in 2016 [31], while in Ghana the CSO among Ghanaian children was stated to be 1.2% [26]. Previous studies have identified factors associated with CSO: including breasting feeding for less than 6 months [32], maternal age [28], socio-economic status [28, 29], wealth status [26], maternal education [29], shorter maternal height [28], and large household size [28].

In Ethiopia, the prevalence of stunting has decreased considerably from 51% in 2005 [33] to 37% in 2019 [34], has continued to decline at an average of more than 1 percentage point per year. Despite these reported improvements, stunting among children is substantially high and remains endemic. At the same time, there is an overall increment in the prevalence of overweight among children in Ethiopia [34, 35]. According to the 2019 Ethiopia Mini Demographic and Health Survey (EMDHS), the prevalence of overweight was 2% and increased from the 2016 EDHS report by 1% [34]. Recent primary studies have also revealed that childhood overweight/or obesity is emerging as a significant childhood public health issue and is consistently increasing in magnitude in Ethiopia [36, 37]. For example, a systematic review by Gebrie and colleagues in 2018, revealed that the combined pooled prevalence of overweight and obesity among children and adolescents in Ethiopia was 11.30% [38].

Although previous studies in Ethiopia have determined the different forms of DBM at the household level [17, 21, 39–41], only a few studies focused on CSO at the individual level [7, 42] and these studies explored CSO using a single snapshot survey. This study builds upon the drawback of the Farah et al. [7] study by combining three EDHSs to examine trends and associated factors of CSO. Hence, this present study aimed to investigate the prevalence, trends, and individual and community-level factors associated with concurrent stunting and overweight or obesity (CSO) among children aged 0–59 months in Ethiopia.

## Methods

### Study setting and data sources

Ethiopia is situated in the Horn of Africa (3°–14° N and 33°–48° E). Amhara, Oromia, Tigray, Benishangul-Gumuz, Somali, Afar, Harari, Southern Nations Nationalities and Peoples (SNNP), Gambella, and two city administration councils (Addis Ababa and Dire Dawa) make up Ethiopia's administrative structure. The study was based on the combined datasets from three consecutive Ethiopia Demographic and Health Surveys (EDHSs) conducted in 2005, 2011 and 2016, a representative sample of the entire population in Ethiopia [33, 35, 43].

### Study design and sampling

The EDHS is a cross-sectional study, which provides a comprehensive overview of population, maternal, and child health issues in Ethiopia with similar sampling methodology applied during data collection in 2005, 2011 and 2016. The EDHS sample was stratified and selected in two stages. In the first stage, enumeration areas (EA) were selected with probability proportional to EA size, with independent selection in each sampling stratum. In the second stage, a fixed number of households per cluster were selected with an equal probability of systematic selection from the newly created household listing [33, 35, 43]. For this study, a total weighted sample of 23,756 children aged 0–59 months were extracted from three surveys and included in the current analysis. The EDHS collected data on the nutritional status of children by measuring the weight and height of children under-five years of age in all sampled households. Children younger than the age of 24 months were measured lying down on the board (recumbent length), while standing heights were measured for older children. These methods have previously been described in the literature [35].

### Variables

#### Outcome variable

The outcome variable was a concurrence of stunting and overweight/obesity (CSO) within the same child. Stunting was defined as height-for-age Z-score (HAZ) below -2SD and overweight/obesity was defined as weight-for-height (or length) z-score (WHZ) above 2 SD from the respective World Health Organization (WHO) 2006 growth standards reference [44] and was dichotomized as co-existence of overweight/obesity and stunting as “Yes”, otherwise, “No”.

#### Independent variables

Potential factors of CSO in children were selected based on previous studies [45–48]. The identified factors were categorized into individual/household and community level factors (Supplementary 1).

### Data analysis

All analyses were carried out using STATA/MP version 14.1 (StataCorp, College Station, TX, USA). Sampling weighting was applied to all descriptive statistics to compensate for the disproportionate allocation of the sample across regions of Ethiopia. The weighting technique is explained in full in the EDHS reports [35]. Given the hierarchical nature of the EDHS data, multilevel logistic regression models were used to determine community and individual-level factors associated with CSO. A multilevel bivariable logistic regression analysis was performed to identify factors associated with the outcome variable. Variables in bivariable multilevel logistic regression analyses with a *p*-value < 0.2 were entered into the multilevel multivariable logistic regression models. The EDHS employed a multistage cluster sampling technique with hierarchical data (i.e., children and mothers were nested within households, and households were nested within clusters). Accordingly, four models were fitted: firstly, the empty model without any explanatory variables was run to detect the presence of a possible contextual effect (*model 1*); the second model was run with individual-level variables (*model II*), the third with community-level variables (*model III*), and the fourth with both individual/household and community-level variables (*model IV*). The intraclass correlation coefficient (ICC) was computed for each model to show the number of variations explained at each level of modeling. An ICC equal or greater than 2% is an indicative of significant group-level variance which is a minimum precondition for a multilevel study design [49]. Model comparisons were performed using the deviance information criterion (DIC) [50, 51]. The model with the lowest DIC was considered the best fit model. Moreover, Akaike Information Criterion (AIC) and the Bayesian Information Criterion (BIC) were used as diagnostics to determine the goodness of fit [52]. Odds ratio (OR) along with 95% confidence interval (CI), were used to estimate the strength of the association. A statistical significance was declared at *p*-value of less than 0.05.

### Operation definition

Concurrent stunting and overweight/obesity (CSO): Children were classified as CSO if they had a HAZ value of < -2SD and their WHZ >  + 2SD simultaneously.

## Results

### Socio-demographic and economic characteristics of the sample

A total of 23,756 children who were born in the last five years preceding the survey were included in the study (4,290 in EDHS-2005; 9,987 in EDHS-2011, and 9,479 in EDHS-2016). Almost half of these children were males (51.1%), and 41.1% of children were in the age group of 36–59 months. Almost three-fourths of the children (73.3%) were currently breastfeeding. Of all the study participants, 69.3% of children were born to mothers with no education, and most children were from rural areas (89.0%) (Table 1).

**Table 1:** Characteristics of the study participants included in the analysis by the individual- and community-level characteristics, EDHS (2005–2016, *n* = 23,756)

**Variables**	**Category**	**Total weighted frequency (n)**	**Weighted percent (%)**
***Individual-level characteristics***			
*** Child factors***			
Sex			
	Male	12,141	51.1
	Female	11,616	48.9
Age (months)			
	< 6	2,470	10.4
	6–11	2,515	10.6
	12–23	4,524	19.0
	24–35	4,489	18.9
	36–59	9,758	41.1
Birth order			
	First born	4,228	17.8
	2–4	10,324	43.5
	5 or higher	9,204	38.7
Birth interval			
	< 33 months	16,550	69.7
	≥ 33 months	7,206	30.3
Size of child at birth			
	Larger	7,478	31.6
	Average	9,593	40.5
	Small	6,601	27.9
Currently breastfeeding			
	Yes	17,421	73.3
	No	6,335	26.7
Received measles (*n* = 19,543)			
	Yes	8,174	41.8
	No	11,369	58.2
Full vaccination (*n* = 19,226)			
	Yes	4,216	21.9
	No	15,010	78.1
Diarrhea (*n* = 23,722)			
	Yes	3,319	14.0
	No	20,403	86.0
Fever			
	Yes	3,965	16.7
	No	19,751	83.3
Children received deworming medication			
	Yes	2,985	14.0
	No	18,301	86.0
***Parental factors***			
Mother's age			
	< 18	175	0.7
	18–24	5,329	22.4
	25–34	12,359	52.0
	≥ 35	5,893	24.8
Mother's education			
	No education	16,469	69.3
	Primary	6,064	25.5
	Secondary	851	3.6
	Higher	373	1.6
Mother's occupation (*n* = 23,657)			
	Not working	12,744	53.9
	Non agriculture	5,179	21.9
	Agriculture	5,733	24.2
Antenatal care (ANC) visit			
	None	8,282	50.8
	1–3	4,171	25.6
	4–7	3,474	21.3
	8 +	362	2.2
Maternal BMI (kg/m^2^)			
	< 18.5	4,774	20.2
	18.5 to 24.9	17,629	74.8
	25 +	1,181	5.0
Any anemia			
	Yes	5,757	24.9
	No	17,393	75.1
Maternal stature (*n* = 23,598)^a^			
	Normal	512	2.2
	Short	8,245	34.9
	Very short	14,840	62.9
Listening to radio			
	Yes	9,060	38.2
	Not at all	14,689	61.8
Watching television			
	Yes	5,324	22.4
	Not at all	18,416	77.6
***Household factors***			
Wealth index			
	Poor	10,762	45.3
	Middle	4,964	20.9
	Rich	8,030	33.8
Household size			
	1–4	5,642	23.7
	≥ 5	18,114	76.3
Type of cooking fuel			
	Clean fuels	405	1.7
	Solid fuels	22,873	98.3
Toilet facility			
	Improved	2,417	10.3
	Unimproved	21,004	89.7
Source of drinking water			
	Improved	10,681	45.6
	Unimproved	12,734	54.4
Household flooring			
	Improved	2,214	9.3
	Unimproved	21,535	90.7
Time to get a water source			
	On-premise	1,752	7.4
	≤ 30 min	13,241	55.7
	31–60 min	5,021	21.1
	> 60 min	3,742	15.8
***Community-level characteristics***			
Residence			
	Urban	2,618	11.0
	Rural	21,138	89.0
Region^b^			
	Large central	21,789	91.7
	Small peripherals	1,384	5.8
	Metropolis	584	2.5
Ecological zone			
	Tropical zone	3,580	15.1
	Subtropical zone	16,703	70.3
	Cool zone	3,473	14.6
Survey year			
	EDHS-2005	4,289	18.1
	EDHS-2011	9,987	42.0
	EDHS-2016	9,479	39.9

^a^Normal/Tall (155 to < 200 cm); Short (145 to < 155 cm), and Very short (< 145 cm); ^b^#: The geographical region of Ethiopia where household heads live. Tigray, Amhara, Oromia, and Sothern Nations Nationalities and Peoples Region (SNNPRs) were categorized under larger central regions; Afar, Somali, Benishangul, and Gambella were under Small peripherals, while Metropolis include Harari, Dire Dawa, and Addis Ababa regions

### Prevalence of stunting, overweight or obesity, and CSO

**Fig. 1:**
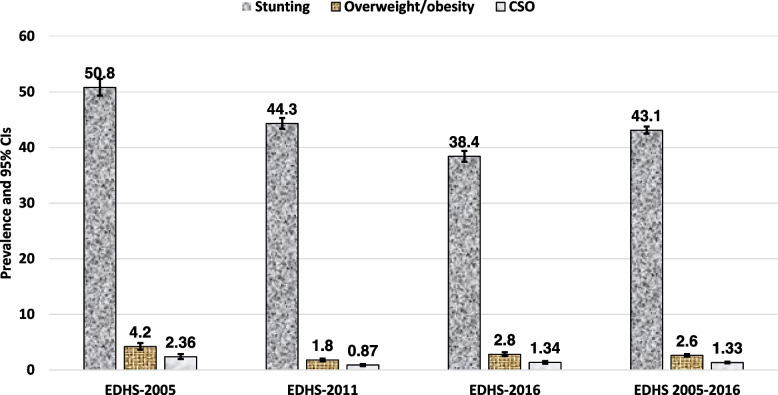
The prevalence and trends of stunting, overweight/obesity and CSO among under-five children in Ethiopia (2005–2016)

The prevalence of stunting and overweight/ obesity among under-five children was found to be 43.12% [95% CI: (42.50, 43.75)] and 2.62% [95% CI: (2.42, 2.83)], respectively. The prevalence of coexistence of stunting and overweight/obesity (CSO) was found to be 1.33% [95% CI: (1.18, 1.48)] (Fig. 1 and Supplementary file 2).

### Trends of stunting, overweight or obesity, and CSO (2005–2016)

**Fig. 2:**
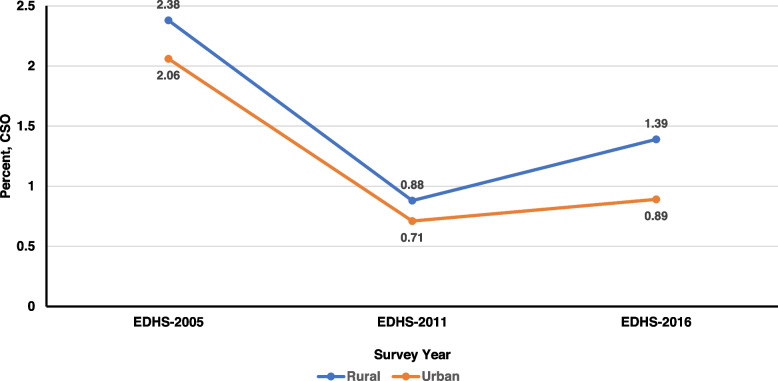
The trend of CSO among under-five children by residence in Ethiopia, EDHS (2005–2016)

**Fig. 3:**
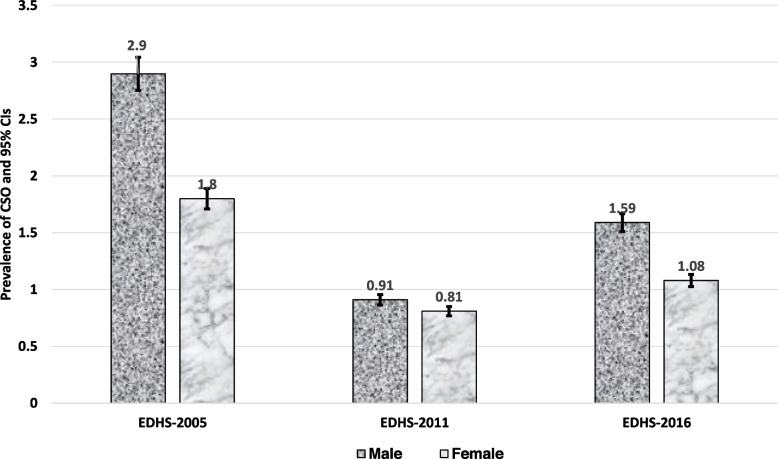
The trends of CSO among under-five children by gender in Ethiopia, EDHS (2005–2016)

**Fig. 4:**
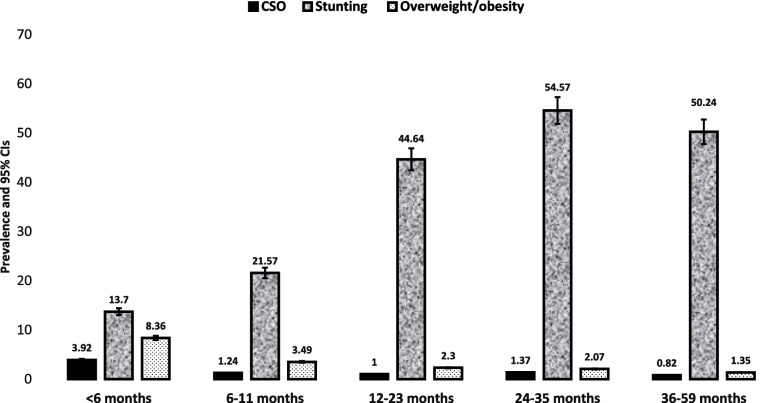
The prevalence of stunting, overweight/obesity and CSO among under-five children by child’s age in Ethiopia, EDHS (2005–2016)

The prevalence of CSO was found to be 2.36% [95% CI: (1.94, 2.85)] in 2005, 0.87% [95% CI: (0.7, 1.07)] in 2011, and 1.34% [95% CI: (1.13, 1.59)] in 2016. There was a significant decrement in CSO prevalence between 2005 and 2016 (Fig. 1). The percentage of stunted children has declined consistently since 2005, from 50.8% [95%CI: (49.3–52.3)] to 38.4% [95%CI: 37.4–39.4)]. The prevalence of overweight/obesity has decreased from 4.2% [95%CI: (3.63–4.84)] to 2.81% [95%CI: 2.49–3.16)] (Supplementary file 2). This decrease is statistically significant because the confidence intervals are not overlapping (Fig. 1). Children in rural areas were more likely than those in urban areas to have CSO throughout the survey years (2.38% versus 2.06% in EDHS-2005, 0.88% versus 0.71% in EDHS-2005, and 1.39% versus 0.89% in EDHS-2016) (Fig. 2). Similarly, between 2005 and 2016, the percentage of male children with CSO was frequently larger than that of females (Fig. 3). Stunting for children under age 5 sharply increases between age 6 and 35 months, and peaks at age 24–35 months. While both overweight/obesity and CSO was prevalent in the first 6 months of age (Fig. 4).

### Factors associated with CSO

In the multilevel bivariable logistic regression analysis, individual-level factors associated with CSO were the age of the child, a child currently breastfeeding, vaccination status, children who received deworming medication, maternal history of ANC visit, watching television, source of drinking water, household flooring, and time to get to the water source. At the community-level, contextual region was associated with CSO (*p*-value of < 0.05) (Table 2).

**Table 2:** Bivariable multilevel logistic regression analysis of individual- and community-level factors associated with concurrent overweight/obesity and stunting (CSO) among children 0–59 months in Ethiopia, EDHS (2005–2016, n = 23,756)

**Variables**	**Number with CSO, %**	**Unadjusted OR, 95%CI**	***p*-value**^**a**^
***Individual-level characteristics***			
***Child factors***			
**Sex**			
Male	187 (1.25)	Ref	
Female	128 (1.01)	0.80 (0.62–1.03)	0.091
**Age (months)**			
< 12	127 (1.97)	Ref	
12–23	43 (0.09)	0.46 (0.31–0.67)**	*p* < 0.001
24–59	144 (0.09)	0.45 (0.34–0.59)**	*p* < 0.001
**Birth order**			
First born	77 (1.33)	Ref	
2–4	139 (1.09)	0.82 (0.59–1.13)	0.225
5 or higher	98 (1.07)	0.80 (0.57–1.23)	0.206
**Birth interval**			
< 33 months	245 (1.16)	1.09 (0.83–1.45)	0.524
≥ 33 months	70 (1.06)	Ref	
**Size of a child at birth**			
Larger	121 (1.20)	Ref	
Average	115 (1.16)	0.96 (0.71–1.29)	0.797
Small	77 (1.02)	0.85 (0.61–1.18)	0.342
**Currently breastfeeding**			
Yes	249 (1.26)	1.51 (1.12–2.03)*	0.007
No	65 (0.84)	Ref	
**Full vaccination**			
Yes	42 (0.82)	Ref	
No	229 (1.36)	1.67 (1.17–2.38)*	0.005
**Diarrhea**			
Yes	40 (0.90)	0.77 (0.52–1.14)	0.190
No	275 (1.17)	Ref	
**Fever**			
Yes	42 (0.85)	0.71 (0.49–1.03)	0.075
No	273 (1.19)	Ref	
**Children received deworming medication**			
Yes	17 (0.39)	Ref	
No	201 (1.02)	2.60 (1.41–4.80)*	0.002
***Parental factors***			
**Mother's age**			
< 18	6 (1.86)	1.65 (0.51–5.34)	0.401
18–24	114 (1.36)	1.20 (0.85–1.70)	0.296
25–34	133 (1.01)	0.89 (0.65–1.22)	0.477
≥ 35	61 (1.14)	Ref	
**Mother's education**			
No education	210 (1.17)	1.09 (0.83–1.44)	0.507
Primary and above	105 (1.06)	Ref	
**Mother's occupation**			
Not working	174 (1.19)	1.30 (0.93–1.83)	0.128
Agriculture	86 (1.22)	1.34 (0.89–2.01)	0.157
Non agriculture	54 (0.91)	Ref	
**ANC Visit**			
None	140 (1.47)	1.54 (1.06–2.22)*	0.022
1–3	73 (1.15)	1.20 (0.78–1.86)	0.405
4 +	41 (0.96)	Ref	
**Maternal BMI (kg/m**^**2**^**)**			
< 18.5	54 (0.95)	Ref	
18.5 to 24.9	232 (1.16)	1.22 (0.89–1.66)	0.216
25 +	22 (1.35)	1.44 (0.86–2.40)	0.162
**Any anemia**			
Yes	78 (1.13)	1.02 (0.77–1.34)	0.911
No	218 (1.11)	Ref	
**Maternal stature**			
Normal	188 (1.14)	Ref	
Short	109 (1.06)	0.93 (0.70–1.22)	0.593
Very short	10 (1.47)	1.29 (0.56–2.94)	0.540
**Listening to radio**			
Yes	101 (0.94)	Ref	
Not at all	213 (1.23)	1.31 (0.99–1.72)	0.057
**Watching television**			
Yes	53 (0.78)	Ref	
Not at all	261 (1.22)	1.55 (1.11–2.19)*	0.011
**Household factors**			
**Wealth index**			
Poor	156 (1.21)	1.07 (0.81–1.41)	0.621
Middle	46 (0.90)	0.79 (0.53–1.20)	0.286
Rich	112 (1.12)	Ref	
**Household size**			
1–4	99 (1.30)	1.22 (0.92–1.60)	0.158
≥ 5	216 (1.09)	Ref	
**Type of cooking fuel**			
Clean fuels	6 (0.08)	Ref	
Solid fuels	307 (1.15)	1.33 (0.59–3.01)	0.492
**Toilet facility**			
Improved	36 (1.15)	Ref	
Unimproved	276 (1.16)	0.96 (0.68–1.36)	0.859
**Source of drinking water**			
Improved	162 (1.34)	Ref	
Unimproved	150 (1.02)	0.72 (0.56–0.93)*	0.013
**Household flooring**			
Improved	14 (0.64)	Ref	
Unimproved	301 (1.22)	2.30 (1.41–3.78)*	0.001
**Time to get a water source**			
On premise	17 (0.79)	Ref	
≤ 30 min	193 (1.25)	1.58 (0.98–2.54)*	0.057
31–60 min	57 (0.97)	1.22 (0.71–2.10)	0.458
> 60 min	47 (1.18)	1.49 (0.88–2.51)	0.132
***Community-level characteristic***			
**Residence**			
Urban	24 (0.97)	Ref	
Rural	289 (1.16)	1.19 (0.83–1.69)	0.345
**Region**			
Large centrals	292 (1.12)	1.53 (1.01–2.34)*	0.048
Small peripherals	19 (1.40)	1.39 (0.89–2.18)	0.142
Metropolis	4 (0.81)	Ref	
**Ecological zone**			
Tropical zone	34 (0.91)	0.88 (0.54–1.46)	0.634
Subtropical zone	241 (1.30)	1.28 (0.80–2.06)	0.298
Cool zone	40 (1.02)	Ref	
**Survey year**			
EDHS-2005	101 (2.46)	2.73 (2.01–3.70)*	*p* < 0.001
EDHS-2011	86 (0.83)	0.92 (0.67–1.26)	0.620
EDHS-2016	127 (0.91)	Ref	

^**^p-value < 0.001, *p-value < 0.05, ^a^The Chi-squared test

Table 3 presents the multivariable multilevel logistic regression analysis results. The odds of having CSO was higher among children who were currently breastfeeding [AOR: 1.64, 95%CI: (1.01–2.72)], children born of an overweight mother [AOR: 2.65, 95%CI: (1.19–5.88)], children living in families having 1–4 household members [AOR: 1.52, 95%CI: (1.02–2.26)], and those included from EDHS-2005 [AOR: 4.38, 95%CI: (2.42–7.95)].

**Table 3:** Multivariable multilevel logistic regression analysis of factors associated with concurrent overweight/obesity and stunting (CSO) among children 0–59 months in Ethiopia, EDHS (2005–2016)

**Variables**	**Null Model (Model I)**	**Model II**^a^	**Model III**^b^	**Model IV**^c^
		**AOR (95%CI)**	**AOR (95%CI)**	**AOR (95%CI)**
***Individual-Level Factors***				
***Child factors***				
**Sex**				
Male		Ref		Ref
Female		0.94 (0.64–1.37)		0.95 (0.64–1.38)
**Age (months)**				
< 12		Ref		Ref
12–23		0.98 (0.57–1.67)		0.95 (0.55–1.63)
24–59		1.34 (0.76–2.35)		1.19 (0.67–2.11)
**Currently breastfeeding**				
Yes		1.63 (1.01–2.66)*		1.64 (1.01–2.72)*
No		Ref		Ref
**Size of child at birth**				
Larger		Ref		Ref
Average		0.83 (0.54–1.29)		0.86 (0.55–1.33)
Small		0.63 (0.38–1.05)		0.68 (0.41–1.14)
**Full vaccination**				
Yes		Ref		Ref
No		0.88 (0.55–1.39)		0.81 (0.51–1.29)
**Diarrhea**				
Yes		0.93 (0.57–1.51)		0.84 (0.51–1.37)
No		Ref		Ref
**Children received deworming medication**				
Yes		Ref		Ref
No		2.47 (1.07–5.72)*		1.79 (0.76–4.25)
***Parental factors***				
**Mother's occupation**				
Not working		1.17 (0.68–1.99)		1.09 (0.63–1.88)
Agriculture		1.57 (0.85–2.88)		1.57 (0.84–2.94)
Non-agriculture		Ref		Ref
**ANC Visit**				
None		1.65 (0.93–2.94)		1.32 (0.72–2.44)
1–3		1.27 (0.68–2.38)		1.29 (0.68–2.46)
4 +		Ref		Ref
**Maternal BMI (kg/m**^**2**^**)**				
< 18.5		Ref		Ref
18.5 to 24.9		1.14 (0.73–1.79)		1.09 (0.69–1.73)
25 +		2.55 (1.17–5.56)*		2.65 (1.19–5.88)*
**Listening to radio**				
Yes		Ref		Ref
Not at all		1.33 (0.84–2.09)		1.56 (0.97–2.49)
**Watching television**				
Yes		Ref		Ref
Not at all		1.06 (0.58–1.94)		0.86 (0.45–1.64)
**Household factors**				
**Household size**				
1–4		1.46 (0.98–2.17)		1.52 (1.02–2.26)*
≥ 5		Ref		Ref
**Toilet facility**				
Improved		Ref		Ref
Unimproved		0.78 (0.42–1.45)		0.70 (0.37–1.33)
**Source of drinking water**				
Improved		Ref		Ref
Unimproved		0.50 (0.33–0.74)*		0.83 (0.53–1.30)
**Household flooring**				
Improved		Ref		Ref
Unimproved		1.78 (0.75–4.17)		1.74 (0.68–4.45)
**Time to get a water source**				
On-premise		Ref		Ref
≤ 30 min		1.49 (0.62–3.57)		1.34 (0.53–3.34)
31–60 min		1.52 (0.59–3.93)		1.49 (0.55–4.00)
> 60 min		1.95 (0.75–5.05)		1.93 (0.72–5.19)
***Community-Level Factors***				
**Residence**				
Urban			Ref	Ref
Rural			0.98 (0.67–1.45)	0.94 (0.42–2.09)
**Region**				
Large central			1.45 (0.92–2.31)	0.99 (0.49–2.00)
Small peripherals			1.82 (1.09–3.03)*	1.64 (0.75–3.58)
Metropolis			Ref	Ref
**Ecological zone**				
Tropical zone			0.99 (0.55–1.77)	0.75 (0.31–1.84)
Subtropical zone			1.50 (0.93–2.43)	1.80 (0.88–3.69)
Cool zone			Ref	Ref
**Survey year**				
EDHS-2005			2.68 (1.96–3.66)**	4.38 (2.42–7.95)**
EDHS-2011			0.92 (0.67–1.26)	1.42 (0.80–2.52)
EDHS-2016			Ref	Ref
**Random effect**				
Variance (SE)	0.1179 (0.0400)	0.2239 (0.0932)	0.1613 (0.0306)	0.2859 (0.0783)
ICC (%)	3.46	6.37	4.67	7.99
**Model comparison**				
LL	-1374.017	-600.17	-1341.17	-578.30
Deviance	2,748.03	1,200.34	2,682.34	1,156.60
AIC	2752.034	1252.344	2700.35	1222.601
BIC	2768.048	1442.109	2772.41	1463.456

NB: *Significant at *p*-value 0.05, **Significant at *P*-value 0.001, *CI* Confidence Interval, *AOR* Adjusted Odds Ratio, *ICC* Intraclass Correlation, ^a^ Adjusted for individual level variables, ^b^ Adjusted for community-level variables, ^c^ Adjusted for individual and community level variables

## Discussion

In this study, we estimated the prevalence and factors associated with childhood (0–59 months) concurrent stunting and overweight/or obesity (CSO) using data from the three waves of EDHS. Our results suggested that the overall prevalence of CSO was 1.33% over the period 2005 to 2016. Based on the full multilevel analysis model, children breastfeeding status, maternal BMI, and household size were identified factors associated with CSO.

The observed prevalence of CSO may have been driven by a rise in children being overweight, coupled with stagnant rates of child stunting in Ethiopia. The prevalence of CSO determined by the current study was in agreement with previous report among Ghanaian children, with a relatively low prevalence of 1.2% [26] and the mother–child pair DBM study from Ethiopia at 1.6 [17]. Additionally the current study indicates a much lower CSO prevalence than reported in previous studies about household-level double burden of malnutrition (DBM) in Ethiopia at 9% [40] and individual level institutional-based study finding in Addis Ababa (Capital city of Ethiopia) at 5.1% [42], 6.6% in Nepal [15], 19.57% in Kenya [53], and 4.7% in Bangladesh [54]. The observed discrepancy may be explained by the study population (as some included mother–child pairs), differences in socio-demographics, and the prevalence of malnutrition between nations. Additionally, our results on CSO at the individual level contradict those of other studies, which took into account the household-level coexistence of an obese mother and a child who is stunted. The occurrence of CSO in Ethiopia is indicative of the nutrition transition that the nation has been going through as a result of changes in eating patterns, high in energy density food intakes, and a decrease in physical activity [14, 55, 56], which has similarly been reported as a global public health problem [56, 57]. The recent evidence from the 2019 Ethiopian Mini Demographic and Health Survey (EMDHS) showed that the prevalence of overweight was 2%, an increase from 1% reported from the 2016 EDHS [34, 35]. Further analysis of EMDHS conducted in 2022 by Gebremichael et al. reported that the overall prevalence of overweight/obesity among under-five children to be 2.14% (95% CI: 1.74–2.53) [58].

The observed prevalence of overweight/obesity among under-five children was lower than the East African regional estimate of 4.59% [14] as well as the prevalence (6.8%) reported in SSA [55]. The current study finding indicate that overweight/obesity was also lower than studies reported in Cameroon (8%) [59], Sierra Leone (16.9%), Comoros (15.9%), and Malawi (14.5%) [55]. This could be because Ethiopia has experienced severe food security issues for many years and is more susceptible to food shortages, disallowing excesses food consumptions that cause overweight and obesity. Moreover, the disparate impacts of the poverty level, lifestyle, socioeconomic status, and food consumption habits of these countries may explain the observed difference. The prevalence of this study, however, was comparable to Senegal (2.0%) in 2011 and Togo (2.6%) in 2014 [55].

The prevalence of stunting in children under five years was very high (43.1%) in Ethiopia between 2005 and 2016. The prevalence of stunting reported in this study is higher than what had been estimated in Rwanda 38% in 2015 [60], Congo 35.2% in 2014 [61], Nigeria 36.7% in 2013 [62], and the East African countries pooled estimate of stunting (33%) [13]. According to the World Health Organization (WHO), the observed prevalence of stunting in Ethiopia is very high (≥ 30%) [63]. This level of childhood stunting should serve as a trigger point for public health intervention.

In this study, children born to overweight/obese mothers were positively associated with CSO. This study found that the odds of CSO was two times higher when mothers were overweight or obese, implying that maternal overweight is associated with poor child health outcomes [64]. Biological, behavioral, environmental, socioeconomic, and demographic factors, and the nutrition transition that has been observed over the past few years in many LMICs, including Ethiopia, may also contribute to the observed association. These changes in dietary patterns seem to be the underlying cause of DBM, where a child could be stunted during the early years and became obese at a later age [5]. Studies have suggest that maternal overweight/obesity is related to having children with higher birth weights [29] and may be linked to child overweight and possible CSO. Evidence suggests that the drivers of these types of malnutrition are shared by biological, environmental, and socioeconomic factors that contribute to the risk of co-occurring conditions [65]. Several pathways could have contributed to and explain these links. Maternal weight gain was significantly higher in households with better food supply and nutrition, which in some cases may well contribute to excessive energy intake and child obesity. Additional, if mothers were exposed to complex factors that contributed significantly to their own weight gain, their children are likely to be exposed to the same complex factors that exacerbate the obesity predisposition during childhood. For instance, a recent meta-analysis identified that maternal pre-pregnancy obesity was significantly associated with child overweight/obesity combined (OR 2.69, 956% CI 2.10–3.46) [66].The impact of maternal obesity extends beyond intrauterine and neonatal life to childhood, adolescence, and adulthood [67]. A cohort study indicated that infants born to obese mothers had a double rate of obesity at age 2 years [68].

The odds of CSO were relatively higher in children living in families with 1–4 household members than those children living in households having five and more 5 household members. It is believed that the size of a family influences the opportunities and resources a child could and received, which in turn could affect the child’s nutrition and development. Studies that have specifically focused on undernutrition have shown the household family size as a major factor associated with child malnutrition [69–71]. A study conducted in eleven Asian countries revealed that the predictive value of household-level factors is much more important for DBM than previously thought [72]. Unlike this study, other studies on concurrent forms of malnutrition have found no links between household size and malnutrition [41]. Thus, more research on CSO in LMICs is needed to determine how CSO relates to the number of household members.

The odds of developing CSO were higher in this study among children currently breastfed. The observed association between breastfeeding patterns and CSO in infants and young children is one of the factors that need to be investigated further to understand better such an association, which is beyond the scope of this study. One probable explanation is that breastfed newborns rely on maternal breastfeeding; under circumstances where food insecurity is a problem as is in Ethiopia, if the mother does not consume enough food and nutrients, her infant may suffer from inadequate milk supply and low-nutrient foods. Because the baby is not getting enough milk, poor milk production can cause problems with nutrients intake, weight gain and poor growth leading to stunting. In addition, if the mother consumes high-energy foods but low-nutrient foods, this may explain the observed association with overweight and obesity in these populations.

In the current study, children from the EDHS-2005 had nearly four times the odds of having CSO than those from the 2016 survey. This finding could be attributed to the difference in the prevalence of stunting and overweight/obesity over the study periods. In our descriptive analysis of CSO, the prevalence was relatively higher in EDHS-2005 than in 2016 (2.36% versus 1.34%). In addition, other factors, such as lifestyle differences across survey periods, may explain the observed finding, due to increasing patterns of nutrition transition over time. For example, the prevalence of stunting was 51% (EDHS-2005), while the prevalence dropped to 38% (EDHS-2016), and the prevalence of severe stunting decreased by more than half (from 28 to 12%) during the period interval of the two surveys [35].

As the study strength, the use of information from a nationally representative population-based survey with a high response rate gave it a stronger statistical power to infer the features of the study population. In addition, reliable estimations were produced using the sampling weight. This study also uses a multilevel logistic regression, which is appropriate for cluster data analysis. Our study has the following limitations: First, the recall bias might have occurred because the birth size and history of infection were reported only by mothers from memory. Second, because this study employed secondary data, it did not account for factors that could affect the occurrence of CSO, such as food security, health problems, and nutrition status during pregnancy. Third, because this study was a cross-sectional design, a cause-and-effect relationship could not be inferred. Fourth, because of the relatively low proportion of CSO in some of the exposure variables, the expected number of observations may be insufficient. As a result, interpretation of some of the findings requires caution.

### Policy implications

In a country where chronic malnutrition has been persistent for centuries, strong policies are needed to address malnutrition and its concurrent forms, such as concurrent stunting and overweight or obesity (CSO). Additionally, addressing the double burden of childhood malnutrition is one of the key factors to achieving the Sustainable Development Goals (in particular Goal 2 and Target 3.4). Our study findings reveal that the burden of CSOs in Ethiopia is increasing. Current national nutrition policies, strategies, and programs need to be tailored for early case identification and management of this concurrent phenomenon. Moreover, given Ethiopia's high prevalence of stunting, strong policies and a commitment to overcoming malnutrition in all forms are required to have a promising impact.

## Conclusion

We found that more than two-fifths of Ethiopian children less than 5 years old were stunted, two in every ten children were overweight or obese, and less than 2% of children had CSO. We found a higher prevalence of CSO among boys rather than among girls, and rural than urban dwellers. Overall, the prevalence of CSO was lower than what has been previously reported in different low-income settings, but the prevalence of CSO was a rising trend between 2011 and 2016. Our results also indicated both individual (i.e. breastfeeding status, maternal BMI, and household size) and community-level factors were associated with CSO. These findings highlight the need for targeted interventions to simultaneously address childhood stunting and overweight or obesity in Ethiopia. Furthermore, to combat CSO, children born to overweight or obese mothers and living in households with multiple household members should be prioritized for earlier interventions. In conclusion, further research is still warranted to address Ethiopia's nutrition transition to tailor public health interventions to address the double burden of childhood malnutrition (undernutrition (stunting) and overweight/obesity in early childhood).

## Supplementary Information

**Additional file 1: Supplementary File 1.** Lists of independent variables included in this study

**Additional file 2: Supplementary File 2.** Prevalence of stunting, overweight/obesity and CSO among in children 0–59 months, EDHS 2005-2016.

## Data Availability

The datasets analysed during the current study are publicly available in the Measure DHS website https://dhsprogram.com after formal online registration and submission of the project title and detail project description.

## Abbreviations

AOR: Adjusted odds ratio
ANC: Antenatal care visits
BMI: Body mass index
CI: Confidence interval
CSO: Concurrent stunting and overweight or obesity
EDHS: Ethiopian Demographic and Health Surveys
SNNP: Southern Nations and Nationalities and People
WHO: World Health Organization
