# Supplementary material for: The coexistence of stunting and overweight or obesity in Ethiopian children: prevalence, trends and associated factors
Source: BMC Pediatr. 2023 May 5;23:218. doi: 10.1186/s12887-023-04037-7 (PMC10163774; doi:10.1186/s12887-023-04037-7)
Supplement: Supplementary file 1 — Additional file 1: Supplementary File 1. Lists of independent variables included in this study [file 12887_2023_4037_MOESM1_ESM.docx]

**Supplementary File 1: Lists of independent variables included in this study**

| **Lists of independent variables** | **Categories** |
| --- | --- |
| ***Individual-level factors*** |  |
| ***Child factors*** |  |
| Sex | 1): Male, 2): Female |
| Age of child (months) | 1): <6, 2): 6-11, 3): 12-23, 4): 24-35, 5): 36-59 |
| Birth order | 1): Firstborn, 2): 2-4, 3): 5 or higher |
| Birth interval | 1): < 33 months, 2): ≥33 months |
| The perceived size of a child at birth | 1): Larger, 2): Average, 3): Small |
| Currently breastfeeding | 1): Yes, 2): No |
| Received measles vaccination | 1): Yes, 2): No |
| Fully vaccinated | 1): Yes, 2): No |
| The child had diarrhea in the last 2 weeks | 1): Yes, 2): No |
| The child had a fever in the last 2 weeks | 1): Yes, 2): No |
| The child receives an iron supplement | 1): Yes, 2): No |
| Received deworming medication in the last 6 months | 1): Yes, 2): No |
| ***Parental factors*** |  |
| Mother's age (years) | 1): <18, 2): 18-24, 3): 25-34, 4): 35-49 years |
| Mother's education status | 1): No education, 2): Primary, 3): Secondary 4): Higher |
| Mother's occupation | 1): Not working, 2): Non-agriculture, 3): Agriculture |
| History of antenatal care (ANC) visit | 1): None, 2): 1-3, 3): 4-7, 4): 8+ |
| Maternal BMI (kg/m2) | 1): <18.5 (underweight), 2): 18.5 to 24.9 (normal), 3): 25 + |
| Maternal anemia | 1): Yes, 2): No |
| Maternal stature | 1): Very short (<145 cm), 2): Short (145 to <155 cm), 3): Normal/Tall (155 to <200 cm) |
| Listening to radio | 1): Not at all, 2): Yes |
| Watching television | 1): Not at all, 2): Yes |
| ***Household factors*** |  |
| Household wealth category | 1): Poor, 2): Middle, 3): Rich |
| Household size | 1): 1-4, 2): ≥ 5 |
| Type of cooking fuel | 1): Clean fuels, 2): Solid fuels |
| Toilet facility | 1): Improved, 2): Unimproved |
| Source of drinking water | 1): Improved, 2): Unimproved |
| Household flooring | 1): Improved, 2): Unimproved |
| Time to get a water source | 1): On premise, 2): ≤ 30 min, 3): 31-60 min, 4): >60 min |
| ***Community Level Factors*** |  |
| Residence | 1): Urban, 2): Rural |
| Region^#^ | 1): Large central, 2): Small peripherals, 3): Metropolis |
| Ecological zones | 1): Tropical zone, 2): Subtropical zone, 3): Cool zone |

#: The geographical region of Ethiopia where household heads live. Tigray, Amhara, Oromia, and Sothern Nations Nationalities and Peoples Region (SNNPRs) were categorized under larger central regions; Afar, Somali, Benishangul, and Gambella were under Small peripherals, while Metropolis include Harari, Dire Dawa, and Addis Ababa regions.
