# Supplementary material for: The coexistence of stunting and overweight or obesity in Ethiopian children: prevalence, trends and associated factors
Source: BMC Pediatr. 2023 May 5;23:218. doi: 10.1186/s12887-023-04037-7 (PMC10163774; doi:10.1186/s12887-023-04037-7)
Supplement: Supplementary file 2 — Additional file 2: Supplementary File 2. Prevalence of stunting, overweight/obesity and CSO among in children 0–59 months, EDHS 2005-2016. [file 12887_2023_4037_MOESM2_ESM.docx]

**Supplementary File 2: Prevalence of stunting, overweight/obesity and CSO among in children 0–59 months, EDHS 2005-2016**

| **Nutritional status** | **EDHS-2005** | | **EDHS-2011** | | **EDHS-2016** | | **Pooled (2000-2016)** | |
| --- | --- | --- | --- | --- | --- | --- | --- | --- |
|  | **Prevalence** | **95%CI** | **Prevalence** | **95%CI** | **Prevalence** | **95%CI** | **Prevalence** | **95%CI** |
| Stunting | 50.9 | 49.4-52.4 | 44.3 | 43.3-45.3 | 38.4 | 37.4-39.3 | 47.3 | 46.8-47.8 |
| Overweigh/obesity | 4.2 | 3.6-4.8 | 1.8 | 1.5-2.0 | 2.8 | 2.5-3.2 | 2.6 | 2.4-2.8 |
| CSO* | 2.3 | 1.9-2.8 | 0.9 | 0.7-1.1 | 1.3 | 1.1-1.6 | 1.3 | 1.2-1.5 |

**There was a significant decrement in the prevalence of stunting and CSO between 2005 and 2016 (Chi square for linear trend p<0.05)*
